# A Short Review on the Electrochemical Performance of Hierarchical and Nitrogen-Doped Activated Biocarbon-Based Electrodes for Supercapacitors

**DOI:** 10.3390/nano11020424

**Published:** 2021-02-07

**Authors:** Glaydson Simões dos Reis, Helinando Pequeno de Oliveira, Sylvia H. Larsson, Mikael Thyrel, Eder Claudio Lima

**Affiliations:** 1 Department of Forest Biomaterials and Technology, Swedish University of Agricultural Sciences, Biomass Technology Centre, SE-901 83 Umeå, Sweden; sylvia.larsson@slu.se (S.H.L.); mikael.thyrel@slu.se (M.T.); 2 Institute of Materials Science, Federal University of Sao Francisco Valley, Petrolina 56304-205, Brazil; helinando.oliveira@univasf.edu.br; 3 Institute of Chemistry, Federal University of Rio Grande do Sul (UFRGS), Av. Bento Gonçalves 9500, Porto Alegre 91501-970, Brazil; profederlima@gmail.com

**Keywords:** biomass resources, pore structure, nitrogen doping, supercapacitors

## Abstract

Cheap and efficient carbon electrodes (CEs) for energy storage systems (ESS) such as supercapacitors (SCs) and batteries are an increasing priority issue, among other things, due to a globally increasing share of intermittent electricity production (solar and wind) and electrification of transport. The increasing consumption of portable and non-portable electronic devices justifies research that enables environmentally and economically sustainable production (materials, processing techniques, and product design) of products with a high electrochemical performance at an acceptable cost. Among all the currently explored CEs materials, biomass-based activated carbons (AC) present enormous potential due to their availability and low-cost, easy processing methods, physicochemical stability, and methods for self-doping. Nitrogen doping methods in CEs for SCs have been demonstrated to enhance its conductivities, surface wettability, and induced pseudocapacitance effect, thereby delivering improved energy/power densities with versatile properties. Herein, a short review is presented, focusing on the different types of natural carbon sources for preparing CEs towards the fabrication of SCs with high electrochemical performance. The influences of ACs’ pore characteristics (micro and mesoporosity) and nitrogen doping on the overall electrochemical performance (EP) are addressed.

## 1. Introduction

The application of biomass residues as the main precursors for producing carbon electrodes (CEs) for energy storage systems (ESS) has been gathering great attention due to its low processing cost and its economic and technical sustainable approaches. ESS, such as batteries and supercapacitors (SCs), are the key to the sustainable development of autonomous electronic devices [1,2].

SCs are promising energy storage devices, and have attracted extensive attention due to their rapid charge and discharge rates, high power density, and long life [3]. Two main mechanisms are responsible for storing energy in supercapacitors: the electrical double layer capacitance (EDLC) and the pseudocapacitance [4,5,6]. The EDLC behavior is a characteristic of carbon derivatives with a high surface area favored by available sites for charge accumulation in a Helmholtz double layer at the electrode–electrolyte interface. The access of charges to these sites is critical for a fast charge–discharge process. These properties limit the energy density in EDLC-based materials. However, pseudocapacitive materials (metals oxides and conducting polymers) are favored by an additional term to the EDLC due to the response in terms of redox reactions, electrosorption, and intercalation that are characterized as fast charge-transfer processes, contributing to the high values for energy density in supercapacitors. The typical drawback in pseudocapacitance candidates is low conductivity (in metal oxides) and poor capacitive retention (fast degradation) in conducting polymers. Consequently, the synergistic interaction between components tends to preserve each material’s advantages, minimizing the drawbacks. The investigation of nanocomposites from materials with outstanding EDLC and pseudocapacitance is a critical topic in the literature.

The development of composites that combine pseudocapacitive behavior and EDLC response in highly porous structures (favored by high surface area and superior electrical conductivity) represents an important strategy to reach electrodes with outstanding electrochemical performance. Zhao et al. [7] reported the development of high energy density and ultrahigh power density from the assembly of electrodes in the form of porous carbon pillars doped with heteroatoms.

Different candidates have been proposed to be applied as EDLC supports (such as graphite, graphene, carbon nanotube, and activated carbon) based on these considerations. However, the scale-up of devices based on these materials is obstructed by the prevailing time-consuming and energy-wasting procedures. Because of this, it is preferable to use easily accessible, renewable, and sustainable materials such as biomass-derived carbon materials [8]. For instance, graphite is a widely used material for energy storage systems fabrication [2]. However, graphite-based devices have reached energy densities close to their theoretical limit, which cannot satisfy the increasing need for higher storage capability, such as electric vehicles and grid energy storage [2,8]. In this sense, to push forward alternative electrodes’ technology there is an imperative need for the design and development of next-generation energy storage devices with similar and/or better electrochemical performances than graphite-derived materials [2,8].

Despite advantages related to the corrosion resistance and ballistic electronic transport in carbon derivative systems (such as biomass-based materials) for use in electrodes, there is a crucial concern relative to the optimization in the energy density–power density of devices in association with reduction of costs in truly scalable systems, due to the typical high-cost processes involving the production of nanoscale-based systems [2].

The production of highly porous carbon materials from biomass is typically based on the carbonization or pyrolysis of material followed by a step of activation (physical or chemical procedure) [9] that confers excellent surface area and porosity degree—the right conditions for EDLC systems. However, as previously described, an adequate condition for the production of high-performance electrodes is based on the combination of Faradaic (pseudocapacitance) and non-Faradaic components (EDLC).

Alternatives to incorporate pseudocapacitance in highly porous carbon derivatives involve the self-doping and artificial doping processes in porous structures. Artificial doping is based on the post-treatment of thermally treated carbon with amines, urea, or phosphoric acid [7]. On the other hand, self-doping is based on direct carbonization/pyrolysis of specific biomass rich in groups that introduce a more homogeneous distribution of heteroatoms into the structure’s bulk. In addition to the pseudocapacitance, these doping elements improve the wettability and the resulting structure’s conductivity.

Given this, the presence of heteroatoms (N, P, S, B, and O) in biomass is preferable, allowing that doped materials can be obtained under thermal treatment and activation. Some examples of intrinsically doped biomass sources are gelatin from animal bones and nitrogen-rich japonica seed [7], winter-jujube (source of O and N) [9], Albizia flowers [10], Cicada slough (source of N, O, S, and P heteroatoms) [10], sharia bambusicola (source of N and Si) [11], boric acid and phosphoric acid (source of B, P, N, and O) [12], and chitosan as an essential source of N and P for self-doped heteroatom structures. It is typical for these structures that they are favored by the most straightforward strategies (one-pot methodology) [13], resulting in cost-effective electrodes and that favor scale-up based strategies. Herein, we summarize the most relevant and recent papers that explore the self-doping of carbon nanostructures with heteroatoms in highly porous carbon derivatives applied as electrodes for supercapacitors, resulting in high surface area materials with good conductivity and superior electrochemical performance. 

## 2. Effect of the Pyrolysis and Experimental Conditions on the AC and CE Properties

The preparation and production of ACs usually occur through both carbonization and activation processes that can be performed either separately in a two-stage process or combined in a single-stage process [14,15,16,17].

Biomass carbonization is performed in the absence of oxygen, generally at temperatures between 400 and 850 °C, to produce AC with useful properties. However, temperatures up to 1200 °C have been reported [14,15,16,17].

**Figure 1:**
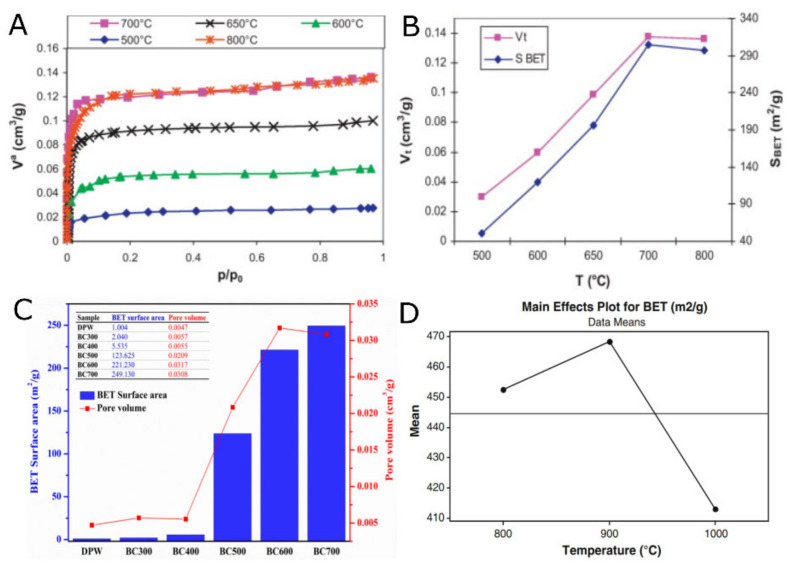
(**A**) N_2_ isotherms of activated carbon (AC) from date pits pyrolyzed at different temperatures. (**B**) Variation of S_BET_ and V_t_ with pyrolysis temperature [22]. (**C**) Effect of T on Brunauer–Emmett–Teller (BET) specific surface area and pore volumes of biochar samples [24]. (**D**) Effect of carbonization temperature on S_BET_ porous surface area [25]. (A) and (B) Ref. [22] is reproduced with permission from Elsevier, 2012. (C) Ref. [24] is reproduced with permission from MDPI (open access), 2019. (D) Ref. [25] is reproduced with permission from Springer Nature (open access), 2014.

Pyrolysis conditions and impregnation by chemical agents have a strong influence on the AC properties. Hence, to obtain AC with characteristics that provide CE with the finest electrochemical properties, it is crucial to find optimum experimental conditions. Generally, the literature indicates that the ACs with the finest properties are produced at high-temperature pyrolysis [14,15,16,17,18] as a consequence of the aromatization process in the carbon chain (enabled at high temperature) [19,20,21]. Bouchelta et al. [22] studied the effects of pyrolysis conditions on the porous structure of ACs made from date pits. The effect of temperature on the AC’s physical properties is shown in Figure 1. Within the experimental range, they found a positive correlation between temperature and S_BET_, which also complies with many other studies [23,24,25]. Figure 1C shows the impact of pyrolysis temperature (0, 300, 400, 500, 600, and 700 °C) on the S_BET_ and the resulting biochars’ pore volumes. The highest S_BET_ and pore volume was achieved for the AC that was pyrolyzed at 700 °C.

However, high pyrolysis temperatures (over 900 °C) negatively impact the final AC characteristics [25]. This effect is mainly associated with longer holding times; the high level of solidification and shrinkage of the carbonaceous matrix results in narrowing or closing the pore entrances, thus reducing the accessible surface area [19,20]. Figure 1D corroborates such a hypothesis, revealing the main effects of pyrolysis temperature on S_BET_ of the ACs—the carbons prepared at 1000 °C exhibited the lowest S_BET_ (Figure 1D).

In addition to pyrolysis temperature, the heating rate, holding time, and chemical activation conditions are essential parameters for the resulting CE performance [26,27,28]. The heating rate has a strong influence on the AC quality; at a very high heating rate, the proportion of the solid product is reduced due to higher volatilization and gas formation; this leads to shrinkage of the AC and reduction in the specific surface area [29,30]. With a lower heating rate, a higher solids yield is obtained [19,20,21].

Generally, a longer holding time results in that secondary char is produced in a reaction between the primary char and volatiles [19,20,22].

The activation of biomass materials is performed to synthesize ACS with high carbon content, large surface area, and pore volume. Additionally, activation can vary or adjust the surface chemical properties and the pore characteristics [31,32,33]. Activation is considered more crucial than carbonization in terms of AC properties, although both are very important and dependents.

There are two activation processes, physical and chemical activation processes. Physical activation involves carbonization of a carbonaceous material followed by activation of the resulting char in the presence of some activating agents such as N_2_, and CO_2_, air, steam, or a mix of these. Otherwise, in the chemical activation process, a chemical agent (ZnCl_2_, H_3_PO_4_, NaOH, or KOH) is applied to activate the biomass while the carbonization occurs [31,32,33]. In physical activation, eliminating a large amount of internal carbon mass is necessary to obtain a well-developed carbon structure. In contrast, in the chemical activation process, all the chemical agents used are dehydrating agents that influence pyrolytic decomposition and inhibit the formation of tar, thus enhancing the yield of carbon and creating active pores and well-developed porosity.

The main factors affecting physical activation include the type of carbon precursors, their particle size, gas flow, heating rate, carbonization time, and carbonization temperature. Chemical activation has the advantage of using low carbonation temperature, short holding time, AC with high carbon content, easy to control the porosity, and elevated SSA [31,33]. On the other hand, it also has some disadvantages: drastic corrosivity and washing process and costly [32,33]. The main factors affecting chemical activation include the type of activating agent, mixing method, the mass ratio of activating agent to carbon precursor, and heating method [31].

## 3. Biomass-Based Electrodes for Supercapacitors

Biomass residues are widely available as plant residues (bamboo, coconut, and peanut shells, petals, soybean, etc.) and raw materials from animals (honeycomb, cattle bone, leather, etc.), and these biomaterials can be used as raw materials for manufacturing CEs for energy storage systems such as supercapacitors.

SCs store and deliver energy by ion adsorption on the surface of the electrically conductive porous CEs. Therefore, CEs exhibiting elevated SSA and developed porosity display quite often better electrochemical properties. 

To date, ACs have been the most used electrode materials in SCs due to their easy preparation methods, very high porosity, and SSA, and excellent electrical conductivity [34,35,36,37,38,39,40,41,42,43,44]. Both the biomass precursor and the preparation method are crucial for the ACs properties and greatly influence the final CEs’ electrochemical features (See Table 1).

In addition to their wide availability and low cost, biomass precursors are suitable for CEs preparation due to the abundance in the functional groups on the resulting CE structures (–COOH, –OH, –NH_2_), and the significant variability in composition, morphology, and textural properties—all very important to reach advantageous electrochemical properties.

Chen et al. [44] used bamboo residues for developing porous carbon electrodes for very efficient SCs through pyrolysis, activation, and heteroatom doping methods. They concluded that the good electrochemical properties of the CE were reached due to the hierarchical porous structure that was obtained by boron and nitrogen co-doping; their specific capacitances and energy densities were 281 and 318 F g^−1^ and 37.8 and 42.1 Wh kg^−1^ in 1 M KOH and 1 M H_2_SO_4_ electrolytes, respectively.

Jiang et al. [40] converted lignocellulosic biomass into highly porous CEs for SCs. The CE microstructures obtained from direct and indirect activation were highly similar. CEs displayed a specific capacitance of 80.9 and 92.7 F g^−1^ at the constant current density of 100 mA g^−1^.

Yakaboylu et al. [41] used Miscanthus grass as the primary precursor for CEs preparation through KOH activation. They concluded that the porosity and chemical characteristics could be controlled by the ratio between biomass/activation agent. Their experimental work resulted in an SSA of 2062 m^2^ g^−1^ and specific capacitance up to 188 F g^−1^ and cycling stability of 85–91% retention (after 1000–2500 cycles) at 0.1 A g^−1^ and specific energy (8.0 Wh kg^−1^) and specific power (377 W kg^−1^) at 0.5 A g^−1^. The authors concluded that the micro/mesopore volume, C/O ratio, and surface chemistry had a considerable influence on the CE’s electrochemical performance due to enhanced electro adsorption, double layer formation, and rapid ion transport.

In another work, Li et al. [9] prepared CE from winter-jujube biomass, obtaining a specific capacitance of 341 F g^−1^ at 0.5 A g^−1^ and a remarkable rate capability (76% at 20 A g^−1^). The capacitance retention was 96.5% after 5000 cycles. Also, CE exhibited an energy density of 30.9 kW h^−1^ at 0.1 A g^−1^ and a power density of 8.9 kW at 5 A g^−1^. These results show that it is possible to use winter-jujube-derived AC as a high-performance CE for SCs.

Hou et al. [30] prepared AC from puffed rice biomass using KOH as an activating reagent. The biomass was pyrolyzed at 850 °C, and an S_BET_ of 3326 m^2^ g^−1^ was reached. The pAC was employed as CE for SCs and exhibited a high specific capacitance of 218 F g^−1^ at 80 A g^−1^ in 6 M KOH and an energy-density of 104 Wh kg^−1^ (53 Wh L^−1^).

These works demonstrate that low-cost and bio-based electrode materials can be used for efficient and eco-friendly manufacturing of innovative energy storage devices with good electrochemical performance.

From Table 1, it is fascinating to note the properties of carrot biomass, reported by Liu et al. [43]. This type of biomaterial provided AC with excellent mechanical and electrochemical properties and an SSA of 682 m^2^ g^−1^. Using aqueous electrolyte (6.0 M KOH) and operating at a potential window between 0 and 1.0 V, the CE displayed a high specific capacitance up to 161 F g^−1^ at 0.2 A g^−1^, a remarkable rate capability (81.8% retention at 20 A g^−1^) and energy densities of 5.6–4.6 Wh kg^−1^ at power densities of 49.8–5884.4 W kg^−1^.

Besides that, when the potential operating window is expanded to 0–1.4 V, the specific capacitance is increased from 161 to 196 F g^−1^ at 0.2 A g^−1^ and 73% retention at 20 A g^−1^ reaching 90% retention after 20,000 cycles, and energy densities of 13.3–9.7 Wh kg^−1^ at power densities of 70.0–8748.3 W kg^−1^.

## 4. Effect of the Pore Structure on the Electrochemical Properties of the CEs

The pore structure plays a significant role in the CEs’ electrochemical properties [30,42,59]. The electrolyte ions can be efficiently transported in small-sized (<1 nm) pores, reaching high charge storage capability at low current density [60,61,62]. However, the electrolyte’s access and flow in small pores are hindered by increasing current density leading to a low ion exchange rate [63,64,65]. However, meso and macropores (pore sizes > 2 nm) give an essential contribution to the rate performance of porous CEs due to the fast transport of solvated electrolyte ions even under high current density [62,66].

Miao et al. [64] reported that a large number of micropores (size between 1 and 2 nm) could lead to a high S_BET_, facilitating the charge separation (due to the available sites for charge accumulation), which affects the overall energy density of the device.

**Figure 2:**
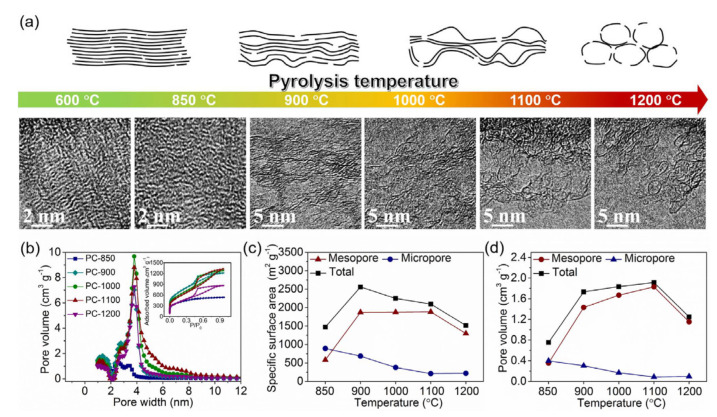
(**a**) Effect of temperature on porous formation illustrated by TEM images. (**b**) Nitrogen adsorption–desorption isotherms (inset) and pore size distributions of ACs. (**c**) S_BET_ and (**d**) pore volume of ACs [36]. Figure reproduced with permission from Elsevier, 2017.

Niu et al. [36] used cattle bones as the sole precursor (without any additional activators and templates) for AC and CE preparation. The biomass was directly carbonized in an Ar atmosphere at different temperatures (T = 600, 850, 900, 1000, 1100, 1200 °C). At 1100 °C, high-defect porous AC was created with an SSA of 2096 m^2^ g^−1^ and a mesopore volume of 1.829 cm^3^ g^−1^ (see Figure 2). These properties resulted in a high electrical conductivity level (5141 S m^−1^), which is beneficial for fabricated electrodes’ electrochemical properties.

The authors concluded that the properties of the AC structures were strongly dependent on the pyrolysis temperature. At higher temperatures (1100 °C), the mesopores were closely connected with the ultrathin pore wall, and some pores even coalesced, which generated a high SSA.

In another work, flaxseed residue was used as raw material to produce AC for CE preparation [37]. The biomass was carbonized at 600, 700, and 800 °C under Ar atmosphere for 2 h. The ACs were further mixed with KOH (ratio of 1:4) and pyrolyzed at 700 °C under Ar atmosphere for 1 h.

**Figure 3:**
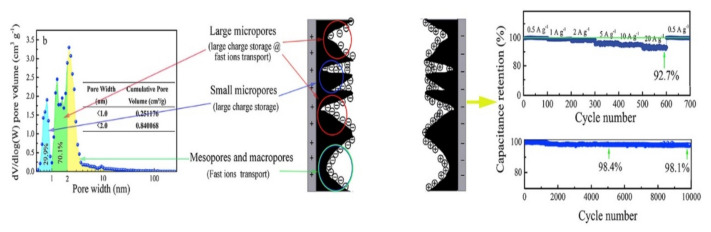
Influence of the pore size on the electrochemical properties of SCs [37]. Figure reproduced with permission from Elsevier, 2020.

The authors reported that AC-flaxseed generated a dominant microporous structure with very high SSA (3230 m^2^ g^−1^) where 70.1% is composed of a large micropore structure (pore size between 1.0 and 2.0 nm) (see Figure 3). Due to the reasonable amount of large micropores, the electrode reached up to 369 and 398 F g^−1^ in KOH and H_2_SO_4_ electrolyte, respectively. These values are justified by a pore size structure that allows a high charge storage capability in response to fast ions transportation. The resulting SCs’ energy density was 61.2 Wh kg^−1^, the power density 468.8 W kg^−1^, and the capacitance retention was over 98.1% after 10,000 charge/discharge cycles (KOH electrolyte).

**Figure 4:**
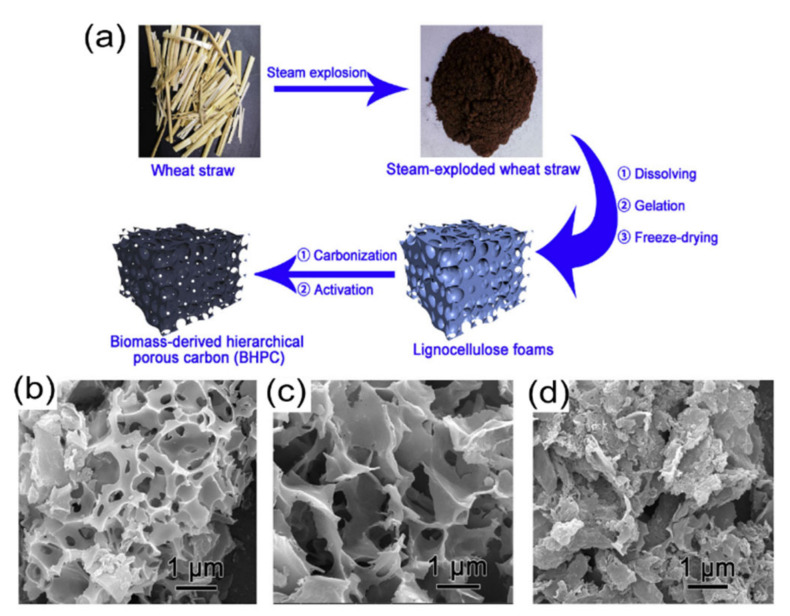
(**a**) Scheme of preparation of the biomass-derived hierarchically porous carbon under different conditions, (**b**–**d**) SEM images of different biomass-derived hierarchically porous carbons. Figure reproduced and adapted from reference [38] with permission from Elsevier, 2014.

Gou et al. [38] used wheat straw to produce hierarchical porous CEs for SCs (see Figure 4). The carbon electrodes were made by chemical activation using KOH at a ratio of 3:1 in weight. Such a Figure shows that all the prepared porous CEs exhibited hierarchical three-dimensional porous structure. In Figure 4b (sample 1), the morphology exhibits a honeycomb-like structure with many pores (420 nm of size). In Figure 4c (sample 2), it is shown a graphene-like structure with wider pores distribution (800 nm of mean size), and in Figure 4d (sample 3), with a resulting layer-stacking structure.

Sample 1 displayed the highest SSA (1063 m^2^ g^−1^) compared to the other samples; however, sample 3 (with SSA of 772 m^2^ g^−1^) presented better electrochemical properties. Sample 1 with the highest S_BET_ did not present the best electrochemical performances because it showed a larger amount of micropores and fewer mesopore contribution. On the other hand, sample 3 presented a high amount of mesopore contributions (see Figure 4). The mesopores provide the high accessibility of a larger surface area for activation process and ion storage and pathways for fast transportation of ions into the structure.

Sample 1 presented micro and mesopore volumes of 0.39 and 0.07 cm^3^ g^−1^, respectively, while sample 2 exhibited between 0.25 and 0.41. It was also related that the pore structure (interconnected three-dimensional nanostructure) provided better efficiency in the ions’ electrolyte transportation—the reason for the outstanding electrochemical performance of SCs. The CEs presented a specific capacitance of 226.2 F g^−1^ at a current density of 0.5 A g^−1^.

## 5. Heteroatom Doping in Porous Electrodes

Heteroatom doping of porous materials is used to reach a high electrochemical performance of AC electrodes for energy storage systems. Among the heteroatoms, nitrogen is known as the most effective doping component. Nitrogen doping is also a feasible method because it changes the structural and physicochemical properties of CEs. Also, nitrogen sources are readily available at a relatively low cost [67,68].

The nitrogen doping process can promote many defects within the AC’s nano- and micro-structures and increase the S_BET_. Besides, N acts as an electron donor that gives more electrons to the delocalized carbon network, increasing the electronic conductivity [69]. The higher the number of N-dopant groups, the higher is the energy density of the CEs. As a consequence of the Faradaic reaction of N groups and the pore walls’ improved wettability, nitrogen doping also increases the overall conductivity, which improves the electrodes’ capacity.

**Figure 5:**
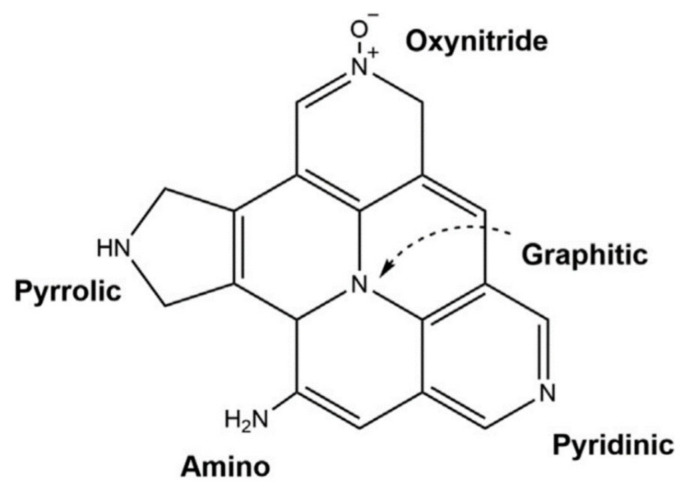
Main nitrogenous contents in carbons.

Usually, the nitrogen doping process is based on five types of N-carbon groups: pyrrolic, pyridinic, aminated functionalities, graphitic (quaternary), and oxynitrides (−NOx) (see Figure 5 [70]- while the graphitic-N bond results in the enhancement of the electron transfer to the structure, the pyridinic-N bonds are weakened by oxygen bonds by adsorption [71,72,73,74].

Many different nitrogenous sources are used in the N-doping process of AC electrodes. Ammonia, urea, melamine, thiourea are the most used ones (see Table 2). The physicochemical properties of these compounds constitute a vital factor for the doping process. For instance, ammonia is very useful for preparing N-porous carbon electrodes due to the following reaction with the carbon source (the biomass): C + NH3 → HCN + H2. The synthesis of hydrogen cyanide and hydrogen provokes a considerable loss of carbon atoms resulting in a more N-doped porous structure [72,73]. Xu et al. [73] stated that the N-doping using ammonia as an N source promoted two main advantages: increasing both S_BET_ and pseudocapacitive contribution for enhanced ion adsorption. 

## 6. Effect of Hierarchically Porous and N-Doped Structures on Electrochemical Performance of Electrodes

As previously reported, the textural features (surface area, pore-volume, pore width distribution) are critically important components in the ACs’ 3-D hierarchical structures. In comparison with corresponding densely packed structures, the porosity facilitates ion adsorption/desorption [75].

Structural combinations of microporous, mesoporous, microporous, and hierarchical porous carbon that might offer high conductivity and characteristic interconnectivity can be produced by different strategies [75,76]. Carbon black, graphene and carbon nanotubes are typical candidates for precursors of highly porous materials [77] due to their fast charge–discharge kinetics, good stability, and environmentally harmless behavior.

To circumvent this, doping with heteroatoms (boron, nitrogen, oxygen, sulfur, and phosphorus) is performed [78], which, as a consequence of the Fermi level shift towards the valence band of the material, improves the electronic conductivity and reinforces the electron transfer.

As a consequence of higher conductivity level, the regular contribution of heteroatom doping on the overall response of highly porous carbon derivatives has been associated with the incorporation of a pseudocapacitive term into the overall electrical double layer capacitance response. This property has been associated with electrodes’ superior performance in surface wettability, high capacity, and cycle stability [77]. In particular, the wettability favors the accessibility (the interfacial interaction) of carbon materials to the electrolyte solution.

The higher accessibility degree of the electrolyte ions to the microporous material structure improves the overall electrochemical, the surface polarity, and the electron-donor affinity [79].

Different strategies have been explored to reach superior electrochemical and textural properties for carbon derivatives. One of the most straightforward procedures that have been applied in the modification of porous carbon structures (incorporation of nitrogen into carbon structure) refers to the post-treatment of porous structure with ammonia gas and urea [79]. Table 2 shows the more relevant results for N-doped biomass-derived electrodes for SCs.

As can be seen, nitrogen-containing compounds such as urea, ammonia, thiourea, polyimide, polypyrrole, melamine, and cyanamide are essential candidates to be incorporated as nitrogen sources for direct pyrolisis of carbon-based sources (see Table 2).

As alternatives for this method of preparation, Zou et al. [79] reported a typical strategy that introduces advantages concerning the scale-up related advantages for the production of porous carbon based on the carbonization of biomass [92]. The authors explored the Juncus (biomass feedstock) as a carbon source to produce N-doped hierarchical porous structures, using ZnCl_2_ as an activation reagent. This process is successfully applied in plenty of carbon source-based materials such as Lycium barbarum L., water hyacinth, and bacterial cellulose [99].

Another possibility refers to the use of potato residues (carbon source) integrated with melamine (nitrogen source) and ZnCl_2_ as a pore-forming component [94].

Yang et al. [75] reported the production of N-doped porous carbon nanofiber aerogel, prepared from the pyrolysis at a high temperature of a carbon source and a nitrogen source (melamine).

In all of these methods, it is observed that an essential step for the production of highly efficient substrates for N-doped supercapacitors refers to the previous activation for the production of higher surface area material with hierarchical opened pores [79].

On the other hand, the reaction with K-species enhances the carbon etching, improving the pore structure of the resulting material, characterizing an essential step for producing materials with desirable electrochemical and textural properties for electrodes with fast adsorption–desorption reactions that connect EDLC and pseudocapacitance reactions.

## 7. Challenges and Future Perspectives

Regarding the AC preparation for energy storage applications, biomass residues as precursors play a crucial role in the development of greener and sustainable CE synthesis process due to its colossal availability and abundance found in every corner of the world.

There is still a gap in the understanding of selecting proper and suitable biomass resources to obtain desirable micro and nanostructures (in terms of pore shapes and sizes) through AC preparation methods, correlating these characteristics on the electrochemical performances. In particular, the intrinsic combination of the high surface area of EDLC candidates with the pseudocapacitive contribution of a second component can be achieved by procedure of nitrogen-doping of resulting structures, in which many aspects need to be evaluated and explored, such as which N-dopant and at which proportion during the AC preparation and applications should be explored and optimized. In contrast, the present studies lack to provide such information. The effective controlling of concentration and their ratio of different doping species are both difficult during the pyrolysis and activation process.

The lack of additional information concerning the electrochemical mechanisms induced by the heteroatom doping effect on biomass-based AC is an essential step toward the complete evaluation of the prominent advantages in terms of the rate capability, chemical stability, cycling performance, and capacitive retention.

The study about a single step experiment for the production of N-doped carbon derivative processes reinforces the possibility of using a low-cost and straightforward procedure to produce all green devices with required properties of energy density and power density to be mutually competitive with conventional batteries and capacitors.

Further development to scale-up the fabrication of biomass AC electrodes to produce suitable and efficient CEs for electrochemical energy devices, including SCs, lithium-ion batteries, etc., is required.

The next step forward for the industry requires the green route to produce materials with outstanding performance and long-range hybrid vehicles’ performance. The toxic compounds of conventional batteries must be urgently substituted by environmentally friendly compounds that explore biomass residues as carbon sources.

## 8. Conclusions

Biomass plays a major role due to the chemical diversity and inherent structures of ACs. The biomass related precursors may lead to the next-generation energy storage devices with similar and/or better electrochemical performances to push forward alternative electrodes’ technology than fossil-derived materials. In this review, the different carbon materials prepared from various biomass precursors with chemical activation have been discussed, and the nitrogen doping effects on electrochemical performances.

The low cost and environmentally friendly aspects of heteroatom-doped structures from biomass are critical advantages for producing outstanding electrochemical components for electrodes of a supercapacitor. The self-doping provided by sources rich in nitrogen introduces adequate pseudocapacitive contribution added to the electrical double layer contribution of highly porous structures to reach high power density and competitive energy density in the state of art supercapacitors. The adequate balance among parameters from the preparation conditions represents the most critical condition for producing materials with outstanding electrochemical performance.

## Figures and Tables

**Table 1:** Electrochemical properties of biomass-based electrodes for supercapacitors (SCs) under different conditions.

Biomass Precursor	Pyrolysis Method and Optimal Condition	SSA (m^2^ g^−1^)	GSC (F g^−1^)	Current Density (A g^−1^)	Specific Energy (Wh kg^−1^)	Power Density (W kg^−1^)	Capacitance Retention (%)	Rate Capability	Electrolyte	Ref
Winter jujube	Pre-carbonized at 400 °C and further pyrolyzed at 800 °C for 2 h with KOH	2286.2	341	0.5	30.9	8.9	96.5	76% at 20 A g^−1^	1 M H_2_SO_4_	[9]
Chinese date	Pyrolyzed at 700 °C and further pyrolyzed at 500, 600, and 700 °C with KOH.	1940.7	518	0.5	18.5	373.8	98.9	-	Na_2_SO_4_	[10]
Chinese date	Pyrolyzed at 700 °C and further pyrolyzed at 500, 600, and 700 °C with KOH.	1940.7	518	0.5	51.3	767.8	90.2	-	Et_4_NBF_4_	[10]
Wild fungus	Pre-carbonized at 400 °C and further pyrolyzed at 800 °C for 2 h with KOH		339	0.5	-	-	98	75% up to 50 A g^−1^	6 M KOH	[11]
Puffed rice	Pre-carbonized at 500 °C and further pyropyrolyzed at 850 °C for 1 h with KOH	3326	218	80	104	53	71	-	6 M KOH	[30]
Seaweed	Carbonized in vacuum at 600–900°C for 3 h and further pyrolyzed at 750 °C for 2 h with KOH	3270	425	0.1	42	390	-	91% at 2.0 A g^−1^	1 M H_2_SO_4_	[42]
Cattle bone	Pyrolyzed at 1100 °C for 1 h and washed HCl	2096	258	5.0	109.9	4400	96.4		EMIM-BF4	[36]
Flaxseed residue	Pyrolyzed at 700 °C and further pyrolyzed at and 700 °C with KOH (1:4).	3230	369	0.5	61.2	468.8	98.1	-	6 M KOH	[37]
Straw residues	Pre-carbonized at 400 °C for 3 h and further pyrolyzed at 600 °C for 1h with KOH	772	226.2	0.5	-	-	78.4	-	6 M KOH	[38]
Bamboo residues	Pyrolyzed at 750 °C for 1 h and further pyrolyzed at 750 °C for 1 h with KOH.	171.5	281	0.2	37.8	97	88	-	6 M KOH	[37]
Carrot biomass	Pre-carbonized at 260 °C for 6 h and further pyrolyzed at 1000 °C for 2 h	682	161	0.2	5.6–4.6	48.8–5884.4	90	81.8% at 20 A g^−1^	6 M KOH	[43]
castor shell powder	AC pyrolyzed at 800 °C with KOH	1527	365	1.0	9.14	500	-	-		[26]
Tea leave residue	A mixture of KOH and tea powder (2:1) and pyrolyzed at 900 °C for 60 min	~912	167	1.0	47.86	1580.72	-	81.42% at 30 A g^−1^	6 M KOH 1 M Na2SO4	[27]
Coconut shell	Biomass/ZnCl_2_ ratio 1:3, then pyrolyzed at 900 °C for 1 h.	1874	268	1.0	11.6	210	99.5	76.9% at 10 A g^−1^	6 M KOH	[28]
Bamboo	Biomass/KOH ratio 1:4 and pyrolyzed at 750 °C.	~172	318	0.2	42.1	210	99.5	76.9% at 10 A g^−1^	1 M H_2_SO_4_	[44]
Shaddock skin	Carbonized at 900 °C for 2 h under Ar and further pyrolyzed at 1200 °C for 1h under vacuum	2327	152	1.0	11	5600	97.6	87% at 100 A g^−1^	EMI TFSI + EMI BF_4_	[45]
Peanut shells	FeCl_3_/MgCl_2_ activated sample at 800 °C	1401.45	~247	1.0	32.7	588.3	96.3	81.8% at 10 A/g	1 M Na_2_SO_4_	[46]
Peanut shells	FeCl_3_/ZnCl_2_ activated sample at 800 °C	1427.81	~186	1.0	22.9	523.8	-		1 M Na_2_SO_4_	[46]
wood sawdust and tannic acid	Potassium chloride + sodium thiosulfate	2650	200	-	47–51	-	97–100	80% at 40 A g^−1^	aqueous electrolyte (H_2_SO_4_)	[47]
wood sawdust and tannic acid		2650	160	-	32–36	140	97–100	75% at 40 A g^−1^	Organic electrolyte	[47]
foxtail grass seeds	Biomass was mixed with NaHCO_3_ and KHCO_3_ (1:1:1) and pyrolyzed at 700 °C for 2 h.		358	0.5	18.2 Wh L^− 1^	-	-	91.2% at 2.0 A g^−1^	6 M KOH	[48]
soybean	Pre-carbonized at 400 °C for 2 h and further pyrolyzed at 750 °C for 2 h with KOH	2251	248	0.1	-	-	98.75	56.7% from 0.1 to 20 A g^−1^	6 M KOH	[49]
Macroalgae	Hydrothermal carbonization + conventional pyrolysis with ZnCl_2_	~2000	202	0.5	7	3000	96	90% at 10 A g^−1^	6 M KOH	[50]
Biomass-based carbon nanofibers			320.3	0.1	30.2	400	70.6	-	6 M KOH	[51]
Dead plant leaves	Pyrolyzed at 1000 °C in for 5 h in argon air	325	345	0.5	43.13	61.34	87.3	-	1 M H_2_SO_4_	[52]
Flagelliforme algae	Pre-carbonized at 400 °C for 1 h and further pyrolyzed at 700 °C for 2 h with KOH activation	2760	283	0.1	22	80	100	-	6 M KOH	[53]
Moringa oleifera stem	Biomass blended with ZnCl_2_ (ratio 1:3) in 50 mL of 2 M FeCl_3_ solution; afterward, pyrolyzed at 800 °C for 2 h under N_2_, for the last, washed with 2.0 M HCl.	2250	283	0.5	11.6	95	82	-	1.0 M Na_2_SO_4_ 1.0 M H_2_SO_4_	[54]
kapok flower	Pre-carbonized at 500 °C for 2 h; soaked in 1.0 M HCl. Afterward, blended with KOH (1:4.5) and further pyrolyzed at 700 °C for 2 h.	1904	286.8	0.5	-	-	97.4	-	6 M KOH	[55]
Tree residues	Biomass pyrolyzed at Tempretarure between 500–700 °C for 10 min under air. Afterward, the pyrolyzed material was chemically activated with a mixture of HNO_3_ and H_2_SO_4_ (HNO_3_:H_2_SO_4_ = 1:3)	616	24	0.25	0.53	51	100	-	1.0 M H_2_SO_4_	[56]
*Syzygium cumini* fruit shells	two-step synthesis: (i) carbonization at 700 °C in N2 atmosphere (ii) CO2 activation at 700 °C in N2 atmosphere.	774	294	0.5	27.22	200	98	-	6 M KOH	[57]
Chrysopogon zizanioides roots	two-step synthesis: (i) carbonization at 700 °C in N2 atmosphere (ii) CO2 activation at 700 °C in N2 atmosphere.	634	253	0.5	16.72	200	98	-	6 M KOH	[57]
Quinoa	carbonized for 120 min at 500 °C and further påyrolysed at 800 °C for 2 h with KOH activation.	2597	254	0.5	22	625	93	75	6 M KOH	[58]
Quinoa	carbonized for 120 min at 500 °C and further påyrolysed at 800 °C for 2 h with KOH activation.	2597	99.2	0.5	9.5	100	93	75	6 M KOH	[58]

GSC—Gravimetric specific capacitance. C.E—Coulombic efficiency.

**Table 2:** −N-doped biomass-derived carbon electrodes for SCs.

Biomass Precursor	N Dopant Precursor	N Content (%)	GSC (F g^−1^)	Current Density (A g^−1^)	Capacitance Retention (%)	Ref
Grape marcs	Urea	2.04	446.0	0.5	Up to 95.1	[80]
Alginic acid	Urea	2.83	324	1.0	Up to 91.5	[81]
Orange peel	Melamine	3.92	168	0.7	-	[82]
Soybean	Ammonia	1.37	243.2	1.0	96.5	[83]
Peach gum	Urea	8.7	426	0.5	97.09	[84]
Glucose	hexamethylenetetramine	-	322	1.0	54.0	[85]
Water chestnut	Melamine	4.89	346	0.5	97.6	[86]
Cellulose	Urea	7.4	570.6	1.0	99.8	[87]
Hierarchical porous carbon	NH_3_.H_2_O + thiourea	7.63	367	0.3	93.7	[88]
Biomass-derived hydrochar	Melamine	4.38	492	0.1	98	[89]
Eucalyptus	Ammonium chloride		359	0.5	92	[90]
Coconut shell	thiourea	4.62	360	0.1	87	[91]
Potato waste	Melamine	6.2	255	0.5	93.7	[92]
Agricultural waste	Urea	2.63	259.5	1.0	95	[93]
Waste lotus stems	Urea		360.5	0.5	96	[94]
Cellulose	Urea	3.0	300	0.5	81	[95]
Lecithin	Urea	9.2	285	0.5	81.3	[96]
Sugarcane bagasse	polypyrrole	3.1	371	0.1	71.5	[97]
Cellulose aerogel	Urea	4.62	225	0.5	81	[98]
